# Supplementary material for: Multi-step retrieval and reasoning improves radiology question answering with large language models
Source: NPJ Digit Med. 2025 Dec 22;8:790. doi: 10.1038/s41746-025-02250-5 (PMC12749912; doi:10.1038/s41746-025-02250-5)
Supplement: Supplementary file 1 — Supplementary Information [file 41746_2025_2250_MOESM1_ESM.pdf]

Multi-step retrieval and reasoning improves radiology question answering with large language models – Supplementary information

Sebastian Wind (1,2), Jeta Sopa (1), Daniel Truhn (3), Mahshad Lotfinia (3), Tri-Thien Nguyen (1,4), Keno Bressem (5,6), Lisa Adams (6), Mirabela Rusu (7,8), Harald Köstler (2,9), Gerhard Wellein (2), Andreas Maier (1,2), Soroosh Tayebi Arasteh* (1,3,7,8)

(1) Pattern Recognition Lab, Friedrich-Alexander-Universität Erlangen-Nürnberg, Erlangen, Germany.
(2) Erlangen National High Performance Computing Center, Friedrich-Alexander-Universität Erlangen-Nürnberg, Erlangen, Germany.
(3) Department of Diagnostic and Interventional Radiology, University Hospital RWTH Aachen, Aachen, Germany.
(4) Institute of Radiology, University Hospital Erlangen, Erlangen, Germany.
(5) Department of Cardiovascular Radiology and Nuclear Medicine, TUM University Clinic, School of medicine and Health, German Heart Center, Technical University of Munich, Munich, Germany.
(6) Department of Diagnostic and Interventional Radiology, TUM University Clinic, School of Medicine and Health, Klinikum rechts der Isar, Technical University of Munich, Munich, Germany.
(7) Department of Radiology, Stanford University, Stanford, USA.
(8) Department of Urology, Stanford University, Stanford, USA.
(9) Chair of Computer Science 10, Friedrich-Alexander-Universität Erlangen-Nürnberg, Erlangen, Germany.

* Correspondence to soroosh.arasteh@rwth-aachen.de

Supplementary Table 1: Characteristics of the RadioRAG dataset used in this study. The RadioRAG dataset combines RSNA-RadioQA and ExtendedQA, as introduced in the original RadioRAG study. Patient demographic information (age and sex) is based solely on the RSNA-RadioQA subset due to missing metadata in ExtendedQA. Each question may be assigned to multiple radiology subspecialties. *Age and sex statistics reflect only the RSNA-RadioQA subset. *Youngest patient was 2 days old. SD: Standard deviation; N/A: Not available.

**Value**	**RadioRAG dataset**
Patient age [years]*<br>Median<br>Mean ± SD<br>Range	 44<br>44 ± 21<br>(0**, 80)
Patient sex [n (%)]*<br>Total<br>Female<br>Male	 80 (100%)<br>37 (46%)<br>43 (54%)
Number of questions per subspecialty [n (%)]	
Total	104 (100%)
Breast Imaging	10 (10%)
Cardiac	10 (10%)
Chest	20 (19%)
CT	35 (34%)
Emergency Radiology	9 (9%)
Gastrointestinal	18 (17%)
Genitourinary	9 (9%)
Head and Neck	10 (10%)
MRI	27 (26%)
Molecular Imaging	11 (11%)
Musculoskeletal	20 (19%)
Neuroradiology	11 (11%)
Nuclear Medicine	13 (12%)
Oncologic Imaging	16 (15%)
Pediatric	8 (8%)
Radiation Oncology	9 (9%)
Ultrasound	10 (10%)
Vascular Imaging	16 (15%)

Supplementary Table 2: Accuracy of 25 language models on the RadioRAG dataset restricted to items with clinically relevant retrieved context. Results are shown for the 48 of 104 questions (46 %) in which the retrieved evidence was judged relevant by expert review (same relevance labels as in **Table 3**). For each model, accuracies are reported for zero-shot prompting and for the RaR framework. Values represent bootstrap means ± standard deviations with 95 % percentile confidence intervals, based on 1,000 shared resampling draws across models and strategies. "Total correct" indicates the number of correct answers out of 48. P-values compare RaR and zero-shot results on paired items using the exact two-sided McNemar test; multiple comparisons were adjusted using FDR.

Model name	Zero-shot			RaR	
	Accuracy (%)	Total correct (n)	P-value	Accuracy (%)	Total correct (n)
Ministral-8B	48 ± 7 [35, 62]	23	0.122	69 ± 7 [56, 81]	33
Mistral Large (123B)	71 ± 7 [56, 83]	34	0.122	88 ± 5 [77, 96]	42
Llama3.3-8B	66 ± 7 [52, 79]	32	0.806	63% ± 7 [48, 77]	30
Llama3.3-70B	81 ± 6 [71, 92]	39	0.566	88 ± 5 [77, 96]	42
Llama3-Med42-8B	68 ± 7 [54, 81]	33	0.539	77 ± 6 [65, 90]	37
Llama3-Med42-70B	71 ± 7 [56, 83]	34	0.122	86 ± 5 [75, 94]	41
Llama4 Scout 16E	77 ± 6 [65, 88]	37	0.146	90 ± 5 [79, 98]	43
DeepSeek R1-70B	86 ± 5 [75, 94]	41	0.748	90 ± 5 [79, 98]	43
DeepSeek R1 (671B)	90 ± 5 [79, 98]	43	0.748	94 ± 3 [85, 100]	45
DeepSeek-V3 (671B)	77 ± 6 [65, 88]	37	0.122	92 ± 4 [83, 98]	44
Qwen 2.5-0.5B	41 ± 7 [29, 54]	20	0.999	44 ± 7 [31, 58]	21
Qwen 2.5-3B	56 ± 7 [44, 71]	27	0.146	71 ± 6 [58, 83]	34
Qwen 2.5-7B	54 ± 7 [40, 69]	26	0.122	75 ± 6 [62, 85]	36
Qwen 2.5-14B	60 ± 7 [46, 75]	29	0.122	79 ± 6 [67, 90]	38
Qwen 2.5-70B	75 ± 6 [62, 88]	36	0.146	90 ± 4 [81, 98]	43
Qwen 3-8B	71 ± 6 [58, 83]	34	0.146	88 ± 5 [77, 96]	42
Qwen 3-235B	83 ± 5 [73, 94]	40	0.566	90 ± 5 [81, 98]	43
GPT-3.5-turbo	54 ± 7 [40, 69]	26	0.148	71 ± 7 [56, 83]	34
GPT-4-turbo	73 ± 7 [58, 85]	35	0.506	81 ± 6 [69, 92]	39
o3	90 ± 4 [81, 98]	43	0.391	96 ± 3 [90, 100]	46
GPT-5	90 ± 4 [79, 98]	43	0.595	94 ± 4 [85, 100]	45
MedGemma-4B-it	50 ± 7 [35, 65]	24	0.391	62 ± 7 [48, 77]	30
MedGemma-27B-text-it	73 ± 6 [60, 85]	35	0.122	94 ± 3 [85, 100]	45
Gemma-3-4B-it	44 ± 7 [29, 58]	21	0.391	56 ± 7 [42, 69]	27
Gemma-3-27B-it	64 ± 7 [50, 77]	31	0.122	86 ± 5 [75, 94]	41

Supplementary Table 3: **RaR-powered responses and retrieved sources for GPT-3.5-turbo on two RSNA-RadioQA questions.** Full answers generated by GPT-3.5-turbo using the RaR framework for the first two questions of the RSNA-RadioQA dataset, along with the most relevant sources retrieved from Radiopaedia. Responses illustrate the model's reasoning and evidence grounding within the RaR pipeline.

Question identifier	LLM's (GPT-3.5-turbo) answer	Most relevant sources
RSNA-RadioQA-Q1	A: BI-RADS 4 - Suspicious abnormality requiring biopsy: BI-RADS 4 lesions are characterized by suspicious findings with an intermediate risk of malignancy. The presence of rim calcifications within a fat-containing mass raises concern for malignancy. Biopsy is generally recommended for BI-RADS 4 lesions, especially if calcifications are suspicious. B: BI-RADS 2 - Rim Calcifications: Rim calcifications in fat-containing masses are typically benign and classified as BI-RADS 2. These calcifications are often associated with benign processes like fat necrosis or oil cysts. The imaging features of a round, circumscribed fat-containing mass with rim calcifications align with a benign diagnosis. C: BI-RADS 3 - Probably benign finding: BI-RADS 3 category is designated for probably benign lesions with a low likelihood of malignancy. Fat-containing lesions with rim calcifications may fall into this category if they demonstrate typical benign features. Short-interval follow-up is recommended for BI-RADS 3 lesions to monitor for any interval changes. D: Oil cyst secondary to fat necrosis: Oil cysts are benign lesions resulting from fat necrosis, often following trauma or surgery. These cysts typically present as fat-containing masses with rim calcifications, consistent with the imaging findings described. The absence of a clear history of trauma or surgery does not exclude the possibility of an oil cyst. Conclude with a Final Answer: Based on the detailed analysis of the imaging findings and the differential diagnosis provided in the report, the most likely diagnosis for the 66-year-old female with a palpable right breast lump at 10 o'clock, characterized by a round, circumscribed fat-containing mass with rim calcifications on mammography, is: D: Oil cyst secondary to fat necrosis. Reasoning: The presence of a round, circumscribed fat-containing mass with rim calcifications in the absence of a clear history of trauma or surgery aligns with the typical imaging features of an oil cyst secondary to fat necrosis. Oil cysts are benign lesions associated with fat necrosis and are characterized by the presence of fat within the lesion and rim calcifications on mammography. This diagnosis is further supported by the benign nature of rim calcifications in fat-containing masses, typically classified as BI-RADS 2. Therefore, considering the imaging findings and clinical context, the most likely explanation for the palpable abnormality in this case is an oil cyst secondary to fat necrosis.	https://radiopaedia.org/articles/breast-imaging-reporting-and-data-system-bi-rads-assessment-category-4, https://radiopaedia.org/cases/suspicious-breast-calcifications-bi-rads-4, https://radiopaedia.org/articles/fat-containing-breast-lesions, https://radiopaedia.org/articles/breast-calcifications, https://radiopaedia.org/cases/screening-mammography-rim-calcifications, https://radiopaedia.org/articles/breast-imaging-reporting-and-data-system-bi-rads-assessment-category-2, https://radiopaedia.org/cases/breast-calcification-bi-rads-2, https://radiopaedia.org/articles/breast-imaging-reporting-and-data-system-bi-rads-assessment-category-3, https://radiopaedia.org/articles/fat-necrosis-breast-2, https://radiopaedia.org/articles/breast-imaging-reporting-and-data-system-bi-rads-2, https://radiopaedia.org/articles/oil-cyst-breast
RSNA-RadioQA-Q2	A: Low-grade fibrosarcoma: Low-grade fibrosarcoma is a rare malignant mesenchymal tumor characterized by spindle cell morphology with a herringbone pattern. Immunohistochemical markers for fibrosarcoma often include vimentin positivity, but specific markers can vary. Beta-catenin nuclear staining is typically absent in fibrosarcomas, which helps differentiate them from other entities. The absence of nuclear beta-catenin staining in the presented case suggests low-grade fibrosarcoma is less likely. B: Phyllodes tumor: Phyllodes tumors are rare fibroepithelial breast tumors with a biphasic pattern of epithelial and spindle cell stromal components. Beta-catenin staining may show nuclear positivity in some cases, reflecting Wnt pathway activation. The clinical presentation of a rapidly growing breast mass in adult women aligns with the patient's characteristics. The presence of spindle cells with nuclear beta-catenin staining in the presented case supports the possibility of a phyllodes tumor. C: Metaplastic carcinoma: Metaplastic carcinoma is a rare primary breast malignancy with both epithelial and mesenchymal components.Beta-catenin expression patterns may show aberrant nuclear localization, associated with Wnt signaling pathway activation. The patient's history of prior radiation exposure aligns with a potential risk factor for metaplastic carcinoma.\n- The spindle cell morphology and nuclear beta-catenin staining in the presented case could be consistent with metaplastic carcinoma. D: Breast fibromatosis: Breast fibromatosis is a rare benign but locally aggressive tumor characterized by spindle cell proliferation and strong nuclear beta-catenin staining.Clinical association with prior radiation exposure matches the patient's history. The spindle cell morphology and nuclear beta-catenin staining in the presented case are in line with breast fibromatosis. Final Answer: D: Breast fibromatosis. Summary of Reasoning: Considering the patient's high risk of breast malignancy due to BRIP1 mutation and prior radiation exposure, the presence of spindle cells with nuclear beta-catenin staining in the breast mass suggests a diagnosis of breast fibromatosis. This rare tumor entity aligns with the clinical presentation, histopathological findings, and the patient's medical history, making it the most likely diagnosis in this case.	https://radiopaedia.org/articles/fibrosarcoma-of-the-breast, https://radiopaedia.org/cases/breast-sarcoma-2?lang=gb, https://radiopaedia.org/articles/phyllodes-tumour-1, https://radiopaedia.org/articles/metaplastic-breast-carcinoma, https://radiopaedia.org/cases/metaplastic-carcinoma-breast, https://radiopaedia.org/articles/fibromatosis-of-the-breast, https://radiopaedia.org/cases/desmoid-fibromatosis-of-the-breast

Supplementary Table 4: **Zero-shot responses from GPT-3.5-turbo on the first 20 questions of the RSNA-RadioQA dataset.** Model-generated answers are shown without RaR. Responses reflect zero-shot inference using only the question text as input.

Question identifier	LLM‘s (GPT-3.5-turbo) answer
RSNA-RadioQA-Q1	The most likely diagnosis is a benign breast lesion, such as a lipoma or fibroadenoma, with associated calcifications.
RSNA-RadioQA-Q2	The most likely diagnosis is desmoid tumor (also known as aggressive fibromatosis).
RSNA-RadioQA-Q3	The most likely diagnosis is Cat Scratch Disease (CSD) with suppurative granulomatous lymphadenitis.
RSNA-RadioQA-Q4	The most likely diagnosis is bilateral invasive ductal carcinoma of the breast, ER positive, PR positive, and HER2 negative, with no lymph node involvement.
RSNA-RadioQA-Q5	The most likely diagnosis is pulmonary rhabdomyosarcoma.
RSNA-RadioQA-Q6	The most likely diagnosis is severe COVID-19 pneumonia.
RSNA-RadioQA-Q7	The most likely diagnosis is pulmonary lymphangioleiomyomatosis (LAM), a characteristic manifestation of Tuberous Sclerosis Complex (TSC).
RSNA-RadioQA-Q8	The most likely diagnosis is a patellar fracture.
RSNA-RadioQA-Q9	The most likely diagnosis is diverticulitis with associated pericolic inflammation and phlegmon formation.
RSNA-RadioQA-Q10	The most likely diagnosis is Hashimoto's thyroiditis.
RSNA-RadioQA-Q11	The most likely diagnosis is orbital sarcoidosis.
RSNA-RadioQA-Q12	The most likely diagnosis is epidural spinal hemangioma.
RSNA-RadioQA-Q13	The most likely diagnosis is a stress fracture of the left femoral neck.
RSNA-RadioQA-Q14	The most likely diagnosis is Kienböck's disease.
RSNA-RadioQA-Q15	The most likely diagnosis is a benign simple cyst of the liver.
RSNA-RadioQA-Q16	The most likely diagnosis is Giant Cell Tumor of Tendon Sheath (GCTTS).
RSNA-RadioQA-Q17	The most likely diagnosis is Alveolar soft part sarcoma (ASPS).
RSNA-RadioQA-Q18	The most likely diagnosis is patellar tendon avulsion fracture.
RSNA-RadioQA-Q19	The most likely diagnosis is benign complicated cysts, given the resolution of the mass with aspiration and the benign nature of the identified cysts on imaging.
RSNA-RadioQA-Q20	The most likely diagnosis is a retroperitoneal teratoma.

Supplementary Table 5: Hallucination and relevance metrics for RaR-powered responses on the internal board-style dataset. Summary of hallucination-related outcomes for the RaR method across all evaluated models on the internal unseen dataset (n = 65). "Context relevant" indicates the proportion of questions with clinically appropriate retrieved content. "Hallucination" refers to incorrect responses despite relevant context. "Correct despite irrelevant context" captures correct answers when the retrieved context was not useful. The final column reports the percentage of questions that were incorrect in zero-shot prompting but answered correctly with RaR.

Model name	Context relevant	Hallucination (relevant context, incorrect response)	Correct despite irrelevant context	Zero-shot incorrect → RaR correct
Ministral-8B	74% (48/65)	6% (4/65)	23% (15/65)	29% (19/65)
Mistral Large (123B)	74% (48/65)	3% (2/65)	25% (16/65)	3% (2/65)
Llama3.3-8B	74% (48/65)	5% (3/65)	20% (13/65)	14% (9/65)
Llama3.3-70B	74% (48/65)	8% (5/65)	25% (16/65)	9% (6/65)
Llama3-Med42-8B	74% (48/65)	15% (10/65)	14% (9/65)	18% (12/65)
Llama3-Med42-70B	74% (48/65)	11% (7/65)	17% (11/65)	14% (9/65)
Llama4 Scout 16E	74% (48/65)	9% (6/65)	26% (17/65)	5% (3/65)
DeepSeek R1-70B	74% (48/65)	9% (6/65)	26% (17/65)	2% (1/65)
DeepSeek R1 (671B)	74% (48/65)	8% (5/65)	25% (16/65)	0% (0/65)
DeepSeek-V3 (671B)	74% (48/65)	5% (3/65)	25% (16/65)	2% (1/65)
Qwen 2.5-0.5B	74% (48/65)	32% (21/65)	17% (11/65)	29% (19/65)
Qwen 2.5-3B	74% (48/65)	9% (6/65)	22% (14/65)	12% (8/65)
Qwen 2.5-7B	74% (48/65)	8% (5/65)	23% (15/65)	17% (11/65)
Qwen 2.5-14B	74% (48/65)	8% (5/65)	25% (16/65)	11% (7/65)
Qwen 2.5-70B	74% (48/65)	5% (3/65)	25% (16/65)	3% (2/65)
Qwen 3-8B	74% (48/65)	11% (7/65)	26% (17/65)	5% (3/65)
Qwen 3-235B	74% (48/65)	9% (6/65)	25% (16/65)	2% (1/65)
GPT-3.5-turbo	74% (48/65)	8% (5/65)	25% (16/65)	22% (14/65)
GPT-4-turbo	74% (48/65)	9% (6/65)	25% (16/65)	15% (10/65)
o3	74% (48/65)	9% (6/65)	26% (17/65)	9% (6/65)
GPT-5	74% (48/65)	12% (8/65)	23% (15/65)	5% (3/65)
MedGemma-4B-it	74% (48/65)	9% (6/65)	25% (16/65)	17% (11/65)
MedGemma-27B-text-it	74% (48/65)	9% (6/65)	25% (16/65)	3% (2/65)
Gemma-3-4B-it	74% (48/65)	11% (7/65)	25% (16/65)	34% (22/65)
Gemma-3-27B-it	74% (48/65)	3% (2/65)	25% (16/65)	15% (10/65)
Average	*74% ± 0*	*9.2% ± 5.5%*	*23.5% ± 3.2%*	*11.8% ± 9.4%*

Supplementary Table 6: Response time comparison between zero-shot and RaR strategies on the internal dataset. Average per-question response times (n=65) are reported in seconds as mean ± standard deviation for both individual models and aggregated model groups. On the internal dataset, a fixed overhead of 5,754.9 seconds per model, corresponding to context generation, was evenly distributed across all questions, contributing approximately 88.5 seconds per question. For time analysis, models were grouped based on parameter scale and architectural characteristics into six categories: the DeepSeek mixture of experts (MoE) group, the large model group (120–250B), the medium-scale group (~70B), the Gemma27B group, the small model group (7–8B), and the mini model group (3–4B). "Absolute difference" denotes the increase in average response time per question introduced by the RaR method, and "Relative increase" refers to the ratio of mean RaR time to mean zero-shot time per group. Final statistics are computed at the group level.

Model / group name	Time			
	Zero-shot (s)	RaR (s)	Absolute difference (s)	Relative increase (times)
**DeepSeek-V3 group**	**65.0 ± 0.0**	**253.5 ± 0.0**	**188.5 ± 0.0**	**3.9 x**
**Large (120 – 250B) group**	**36.9 ± 16.8**	**216.7 ± 73.0**	**179.8 ± 72.3**	**5.9 x**
Llama4 Scout 16E	36.3 ± 20.1	133.2 ± 20.4	96.8 ± 20.0	3.7 x
Mistral Large	20.3 ± 10.1	249.1 ± 78.9	228.8 ± 71.2	12.3 x
Qwen 3-235B	54.0 ± 28.7	267.8 ± 89.7	213.9 ± 79.2	5.0 x
**Medium (≈ 70B) group**	**36.5 ± 6.8**	**163.2 ± 22.7**	**126.6 ± 26.2**	**4.5 x**
DeepSeek R1-70B	41.8 ± 23.7	173.1 ± 45.6	131.2 ± 41.4	4.1 x
Llama3-Med42-70B	36.8 ± 18.1	133.2 ± 21.6	96.5 ± 20.8	3.6 x
Llama3.3-70B	40.6 ± 20.7	160.0 ± 34.8	119.4 ± 31.3	3.9 x
Qwen 2.5-70B	26.9 ± 14.9	186.4 ± 39.7	159.4 ± 35.3	6.9 x
**Gemma 27B group**	**53.7 ± 36.9**	**161.1 ± 54.3**	**107.4 ± 17.4**	**3.0 x**
Gemma-3-27B-it	27.6 ± 13.2	122.7 ± 17.0	95.1 ± 16.0	4.4 x
MedGemma-27B-text-it	79.8 ± 41.6	199.5 ± 53.3	119.7 ± 49.8	2.5 x
**Small (7 – 8B) group**	**10.3 ± 15.3**	**104.9 ± 11.0**	**94.6 ± 6.9**	**10.2x**
Llama3-Med42-8B	2.4 ± 1.1	94.1 ± 2.5	91.7 ± 2.1	38.5 x
Llama3.3-8B	5.9 ± 3.1	99.8 ± 5.5	93.8 ± 4.9	16.8 x
Ministral-8B	2.9 ± 1.2	100.9 ± 5.8	98.0 ± 5.3	34.4x
Qwen 2.5-7B	2.9 ± 1.3	106.8 ± 4.6	104.0 ± 4.0	37.2 x
Qwen 3-8B	37.5 ± 20.8	123.0 ± 20.7	85.5 ± 20.7	3.3 x
**Mini (3 – 4B) group**	**7.7 ± 3.8**	**105.3 ± 6.5**	**97.6 ± 9.1**	**13.7 x**
Gemma-3-4B-it	12.0 ± 5.0	100.2 ± 5.7	88.1 ± 5.6	8.3 x
MedGemma-4B-it	6.3 ± 3.6	112.6 ± 14.5	106.3 ± 15.7	18.0 x
Qwen 2.5-3B	4.8 ± 2.3	103.0 ± 3.8	98.2 ± 3.3	21.4 x
Average	*35.0 ± 22.9*	*167.5 ± 59.4*	*132.4 ± 41.7*	*6.9 ± 4.2 x*

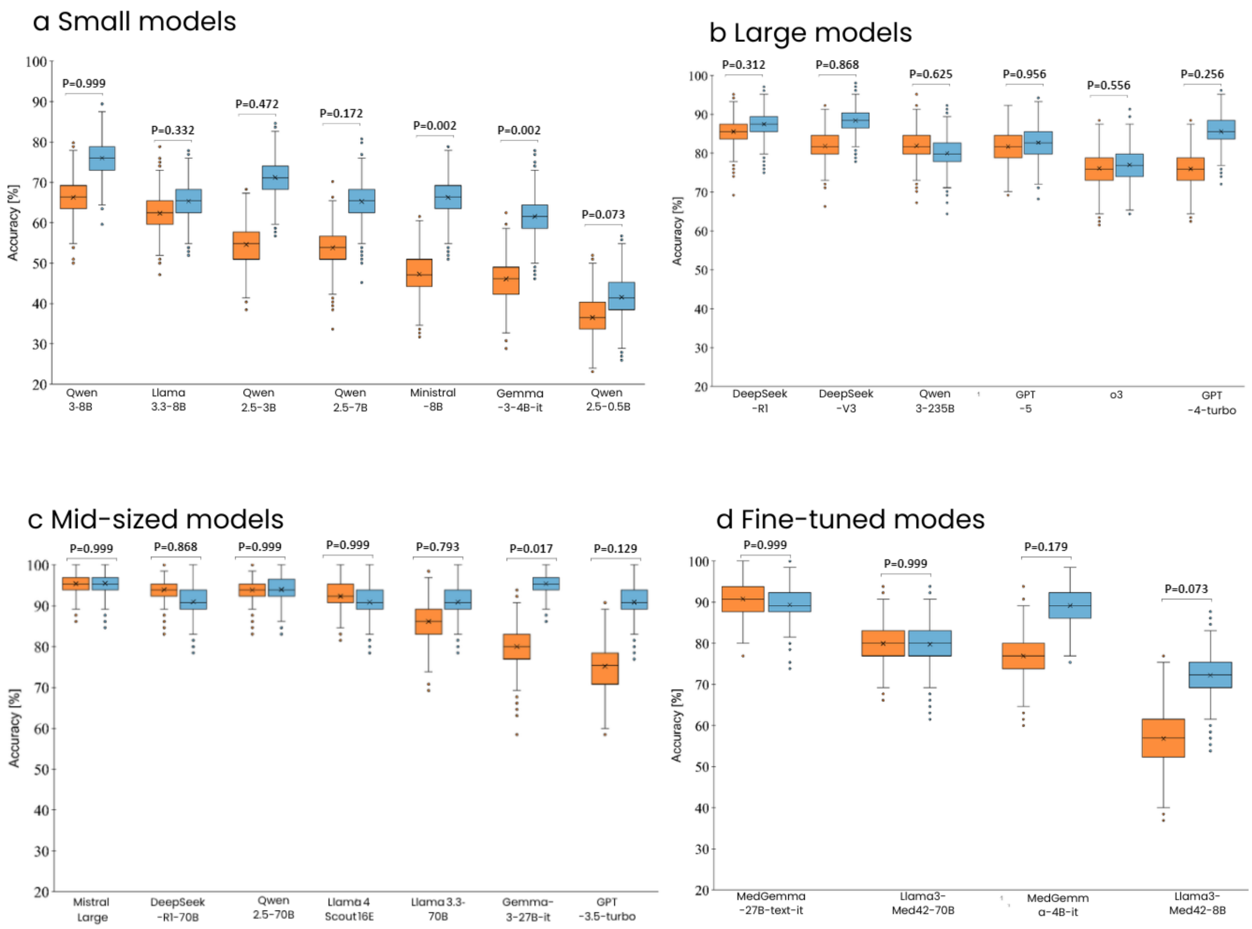

Supplementary Figure 1: Comparative accuracy distributions for zero-shot versus RaR strategies across model groups on the internal dataset. Accuracy results are shown for **(a)** small-scale models (Ministral-8B, Gemma-3-4B-it, Qwen 2.5-7B, Qwen 2.5-3B, Qwen 2.5-0.5B, Qwen 3-8B, Llama 3-8B), **(b)** large models (o3, GPT-5, DeepSeek-R1, Qwen 3-235B, GPT-4-turbo, DeepSeek-V3), **(c)** mid-sized models (Mid-Sized Models: GPT-3.5-turbo, Llama 3.3-70B, Mistral Large, Qwen 2.5-70B, Llama 4 Scout 16E, Gemma-3-27B-it, DeepSeek-R1-70B), **(d)** and medically fine-tuned models (MedGemma 27B-text-it, MedGemma 4B-it, Llama3-Med42-70B, Llama3-Med42-8B). comparisons were performed on the internal benchmark dataset (n =65). Boxplots display accuracy (%) distributions (n = 1 000) for zero-shot (orange) and RaR (blue): boxes span Q1–Q3, central line is the median (Q2), whiskers extend to 1.5×IQR and dots mark outliers. P-values were calculated between each pair's accuracy values for each model using McNemar's test on paired outcomes relative to RaR and adjusted for multiple comparisons using the false discovery rate. A p-value < 0.05 was considered statistically significant.

Supplementary Note 1

Diagnostic abstraction and retrieval preprocessing

To enable structured, multi-step reasoning within the RaR (Retrieval and Reasoning) framework, we implemented a preprocessing step focused on diagnostic abstraction. This step ensured that retrieval operated on concise, clinically meaningful summaries of each question rather than on the full text, thereby improving retrieval precision and interpretability.

For every question in the RadioRAG dataset, we generated a short, comma-separated summary of its key diagnostic elements, typically including patient demographics, major imaging findings, clinical symptoms, and distinguishing modifiers (e.g., lesion type, location, or contrast pattern). These structured summaries served as retrieval queries to identify the most relevant clinical reports from Radiopaedia. To produce these abstractions, we used the Mistral Large (123B) model after comparing it against GPT-4o-mini and LLaMA-2-70B. Alternative models often produced verbose, incomplete, or redundant summaries, whereas Mistral Large consistently yielded concise, clinically faithful concept lists with strong semantic alignment to the original diagnostic intent. Its balance of brevity and coverage made it particularly effective for retrieval guidance within RaR.

Representative cases

Case 1
Question: “A 45-year-old female with active HIV infection presented for a routine screening mammogram, which showed enlarged axillary lymph nodes but no other abnormalities. What is the most likely diagnosis?”
Keywords generated by Mistral Large: 45-year-old female, active HIV infection, routine screening mammogram, enlarged axillary lymph nodes, no other abnormalities.

Case 2
Question: “A 15-year-old boy with scoliosis and recurrent nosebleeds has an incidental right middle lobe nodule.”
Keywords generated by Mistral Large: 15-year-old male, no significant past medical history, scoliosis, frequent nosebleeds, dextroconvex thoracic scoliosis, incidental right middle lobe nodule.

Case 3
Question: “A 61-year-old man with a painless, exophytic chest-wall mass of 18 months’ duration that bleeds occasionally and shows spindle cells in a storiform pattern on biopsy.”
Keywords generated by Mistral Large: 61-year-old man, painless fungating chest wall mass, 18 months' duration, occasional bleeding, vision impairment, spindle cells in storiform pattern, superficial exophytic soft tissue mass, no deep invasion.

Case 4
Question: “Strongly hyperdense lesions are seen in pulmonary arteries after vertebroplasty. What is the most likely diagnosis?”
Keywords generated by Mistral Large: hyperdense lesions in segmental pulmonary arteries of right lower lobe on non-contrast CT, history of vertebroplasty.

Diagnostic abstraction improved retrieval precision by reducing irrelevant matches caused by verbose or narrative phrasing. When applied across the full RadioRAG dataset, the retrieval pipeline incorporating these abstractions achieved clinically relevant context in 46% (48/104) of questions, as reported in the main text (**Table 3**). Although direct pre-abstraction retrieval rates were not logged, qualitative inspection confirmed that keyword-based queries produced more focused and semantically appropriate Radiopaedia matches, particularly for long, multi-clause questions. Overall, diagnostic abstraction provided a structured interface between natural-language questions and evidence retrieval, translating complex diagnostic phrasing into standardized clinical concept lists and enabling RaR to achieve more precise, interpretable, and contextually grounded reasoning.

Supplementary Note 2

Case studies: correct answers despite irrelevant or noisy retrieved context

For each question in the RadioRAG dataset, a board-certified radiologist labeled the retrieved context as either clinically relevant or irrelevant, with the same label applied across all models for that item. Representative cases from the RaR runs of DeepSeek-R1 and GPT-3.5-turbo illustrate three characteristic reasoning patterns: context-independent correctness, where the model provides a correct answer despite irrelevant context; context-dependent grounded correctness, where the correct answer is supported by relevant evidence; and reasoning-shortcut errors, where the model gives an incorrect answer despite access to correct contextual information. These examples correspond to real RadioRAG items and are identified by their respective question IDs.

Case 1 – Context-independent correctness
Question: "Strongly hyperdense lesions are noted in the segmental pulmonary arteries of the right lower lobe on a non-contrast CT of the thorax. The patient has a history of vertebroplasty. What kind of lesions could that be?"
Reference answer: Pulmonary cement embolism
Retrieved context: Irrelevant
Model outcomes (RaR):

- DeepSeek-R1 — Correct
- GPT-3.5-turbo — Correct

Both models inferred the correct diagnosis from internal reasoning ("hyperdense pulmonary artery lesion + vertebroplasty → cement embolism"), without relying on the noisy retrieval. This exemplifies context-independent correctness, where accuracy arises from internal knowledge rather than the retrieved evidence.

Case 2 – Grounded correctness
Question: "A 61-year-old man presented with a painless, large, fungating chest wall mass of 18 months\u2019 duration that occasionally bled and impaired his vision of his feet. There were no systemic symptoms and staging CT of the chest, abdomen, and pelvis showed no other lesions. Biopsy revealed spindle cells in a storiform pattern infiltrating fat. MRI confirmed a superficial exophytic soft tissue mass confined to cutaneous and subcutaneous planes without deep invasion. What is the most likely diagnosis?"
Reference answer: Dermatofibrosarcoma protuberans
Retrieved context: Relevant
Model outcomes (RaR):

- DeepSeek-R1 — Correct
- GPT-3.5-turbo — Correct

This case shows an ideal evidence-grounded success. Both models effectively used the retrieved pathology and imaging cues to confirm the correct diagnosis.

Case 3 – Reasoning shortcut error
Question: “A 15-year-old male with no significant past medical history presents to orthopedic clinic for evaluation of scoliosis. An abnormality is seen on scoliosis films. Review of systems is positive for frequent nosebleeds. Frontal and lateral views of the spine show dextroconvex thoracic scoliosis with an incidental nodule in the right middle lobe. What is the most likely diagnosis?”
Reference answer: Pulmonary varix
Retrieved context: Relevant
Model outcomes (RaR):

- DeepSeek-R1 — Incorrect (predicted Pulmonary AVM)
- GPT-3.5-turbo — Incorrect

Both models defaulted to the familiar association “epistaxis → AVM,” ignoring the key exclusion in the evidence. This represents a reasoning-shortcut error: failure to integrate a disconfirming retrieved fact.

These representative examples illustrate distinct patterns of reasoning under retrieval-augmented conditions. Some models can produce accurate answers even when retrieval is clinically irrelevant (Case 1), demonstrating robustness of internal medical knowledge. When evidence is relevant, RaR supports properly grounded reasoning by enabling structured integration of the retrieved content (Case 2). However, overreliance on prior associations can still lead models to disregard disconfirming evidence, resulting in errors despite correct retrieval (Case 3). Together, these cases show that RaR improves evidence use overall, yet retrieval relevance alone does not guarantee reasoning correctness, a key motivation for explicit grounding and auditing in clinical LLM pipelines.

Supplementary Note 3

Error analysis

To better understand the reasoning patterns underlying model performance, we performed a qualitative error analysis across representative cases in the RadioRAG benchmark. Each question–answer pair was manually reviewed by a board-certified radiologist (TTN) to identify the dominant reasoning failure or success type. Errors were categorized into three principal types: reasoning shortcut errors, context integration errors, and context independence errors.
Reasoning shortcut errors occurred when models defaulted to familiar diagnostic associations instead of verifying all relevant details in the retrieved evidence.

- Example: In RSNA-RadioQA-Q59, all 25 models misdiagnosed a 15-year-old with frequent nosebleeds and a small lung nodule as having a pulmonary arteriovenous malformation. The report clearly described a pulmonary varix lacking an artery-to-vein connection, which should have excluded the diagnosis. Models relied on pattern familiarity ("nosebleeds → AVM") rather than applying the exclusion rule provided in the evidence. The radiologist made the same initial error without retrieval but corrected it after reviewing the RaR-retrieved report, confirming that the issue was reasoning, not data availability.

Context integration errors reflected failures to synthesize multiple correct elements into a single, coherent diagnosis.

- Example: In RSNA-RadioQA-Q5, which described a two-year-old with a large lung mass, most models focused narrowly on biopsy findings suggestive of a muscle-type tumor and ignored age and imaging clues indicating pleuropulmonary blastoma. The human expert correctly integrated these details to reach the right answer, demonstrating that the provided evidence was sufficient.

Context independence errors involved correct answers derived without meaningful use of the retrieved evidence.

- Example: In RSNA-RadioQA-Q88, the retrieved report discussed right-heart strain but also contained unrelated cardiac descriptions. Despite this, most models and the radiologist correctly diagnosed pulmonary embolism based on imaging clues such as right-ventricular enlargement, septal bowing, and contrast reflux. Likewise, in RSNA-RadioQA-Q97, all models correctly identified pulmonary cement embolism even though the retrieved report was classified as irrelevant. In both cases, models solved the question using internal medical knowledge rather than integrating the retrieved text, analogous to answering an open-book test without consulting the book.

Together, these analyses reveal that RaR mitigates reasoning shortcut and integration errors by enforcing structured, evidence-based reasoning, but context independence persists when retrieval adds noise rather than clarity. These findings highlight the need for future methods that not only improve factual accuracy but also ensure faithful use of supporting evidence. Overall, RaR contributes to more transparent and evidence-grounded diagnostic reasoning, even when retrieval context is imperfect.

Supplementary Note 4

Sensitivity/precision analysis and subgroup comparisons

To contextualize non-significant findings and quantify precision, we performed subgroup-level paired analyses across models. For each dataset (RadioRAG, n = 104; internal n = 65), models were grouped as small, mid-sized, large, and clinically fine-tuned, consistent with the main manuscript.

The small subgroup included Ministral-8B, Gemma-3-4B-it, Qwen 2.5-7B, Qwen 2.5-3B, Qwen 2.5-0.5B, Qwen 3-8B, and Llama-3-8B. Mid-sized models comprised GPT-3.5-turbo, Llama 3.3-70B, Mistral Large, Qwen 2.5-70B, Llama 4 Scout 16E, Gemma-3-27B-it, and DeepSeek-R1-70B. Large-scale models included DeepSeek-R1, DeepSeek-V3, o3, Qwen 3-235B, GPT-4-turbo, and GPT-5. Clinically fine-tuned models consisted of MedGemma-27B-text-it, MedGemma-4B-it, Llama3-Med42-70B, and Llama3-Med42-8B. Qwen 2.5-14B was excluded as it did not align clearly with any predefined category.
Within each subgroup, mean accuracies under zero-shot and RaR conditions were computed, and paired differences were analyzed across models using two-sided paired t-tests. Reported statistics include the mean difference, its 95 % CI (t-based), p-value, and Cohen's dz.

On the RadioRAG dataset, RaR improved mean accuracy most notably for small models (+11.43 percentage points (pp), p = 0.002) and mid-sized models (+7.57 pp, p = 0.001), with a smaller and statistically non-significant effect in large models (+3.00 pp, p = 0.147). Clinically fine-tuned models also showed a consistent and significant gain (+8.75 pp, p = 0.001). On the internal generalization dataset, RaR produced a large and significant improvement in small models (+14.71 pp, p = 0.010), with positive but non-significant trends for mid-sized (+4.57 pp, p = 0.174) and clinically fine-tuned (+6.25 pp, p = 0.238) subgroups, and no measurable difference in large models (−0.17 pp, p = 0.953).
Overall, these subgroup analyses indicate that RaR's performance gains are most pronounced and statistically robust among smaller and mid-sized models, consistent with the main-text results. The lack of significance in other groups likely reflects limited sample size rather than the absence of true effect.

Supplementary Table N4.1: **Subgroup accuracy comparison between zero-shot prompting and RaR on the RadioRAG dataset (n=104).** The table summarizes subgroup-level mean accuracies and paired differences between zero-shot prompting and RaR. Reported metrics include the number of models per subgroup, mean accuracies, mean paired differences, standard deviations of differences, 95 % confidence interval (CI) limits, p-values from two-sided paired t-tests, and Cohen's dz effect sizes.

Subgroup	Small	Mid-sized	Large	Finetuned
Number of models	7	7	6	4
Zero-shot mean (%)	52.43	70.57	80.67	66.50
RaR mean (%)	63.86	78.14	83.67	75.25
Mean difference (%)	+11.43	+7.57	+3.00	+8.75
Standard deviation of differences (%)	5.97	3.26	4.29	1.50
95% CI low	5.92	4.56	-1.50	6.36
95% CI high	16.94	10.59	7.50	11.14
P-value	0.002	0.001	0.147	0.001
Cohen dz	1.91	2.32	0.70	5.83

Supplementary Table N4.2: **Subgroup accuracy comparison between zero-shot prompting and RaR on the internal dataset (n=65).** The table summarizes subgroup-level mean accuracies and paired differences between zero-shot prompting and RaR. Reported metrics include the number of models per subgroup, mean accuracies, mean paired differences, standard deviations of differences, 95 % confidence interval (CI) limits, p-values from two-sided paired t-tests, and Cohen's dz effect sizes.

Subgroup	Small	Mid-sized	Large	Finetuned
Number of models	7	7	6	4
Zero-shot mean (%)	69.57	88.43	90.00	76.25
RaR mean (%)	84.29	92.57	89.83	82.50
Mean difference (%)	+14.71	+4.57	-0.17	+6.25
Standard deviation of differences (%)	10.42	7.85	6.55	8.50
95% CI low	7.90	−2.67	−7.06	-7.28
95% CI high	21.53	11.82	6.73	19.78
P-value	0.010	0.174	0.953	0.238
Cohen dz	1.41	0.58	-0.03	0.74

Supplementary Note 5

This file contains the complete internal dataset, consisting of 65 expert-authored radiology questions.

Question Identifier: Internal-Q1

Question: A 45-year-old patient with Child-Pugh B cirrhosis undergoes gadoxetic acid-enhanced MRI. Multiple nodular lesions are identified with varying enhancement patterns. Which combination of signal characteristics, enhancement kinetics, AND ancillary features provides the highest specificity (>95%) for differentiating an early well-differentiated hepatocellular carcinoma from a high-grade dysplastic nodule, considering the LI-RADS v2018 criteria?

Options:

A) T1 hyperintense (due to glycogen/fat), T2 hypointense to isointense, arterial phase hyperenhancement with portal venous washout, AND restricted diffusion on DWI with ADC values $<1.0 \times 10^{-3}$ mm²/s
B) T1 isointense, T2 moderately hyperintense, no arterial enhancement, portal venous hypoenhancement, normal diffusion
C) T1 hypointense, T2 hyperintense, arterial hyperenhancement without washout, facilitated diffusion
D) T1 hyperintense, T2 isointense, homogeneous enhancement in all phases, no diffusion restriction
E) T1 hypointense, T2 hypointense, complete absence of enhancement in all phases, marked diffusion restriction

Solution (Reference Standard Answer): Option A

Question Identifier: Internal-Q2

Question: When planning Gamma Knife stereotactic radiosurgery for 5 brain metastases (8-12mm diameter each) using a prescribed marginal dose of 20 Gy at the 50% isodose line, multiple dosimetric parameters must be optimized. Which statement regarding dose distribution, normal tissue constraints, and radiobiological considerations is FALSE?

Options:

A) The Paddick conformity index should ideally be between 0.9-1.1, while the gradient index should be <3.0 to minimize the intermediate dose spillage
B) The V12Gy (brain volume receiving ≥12 Gy) should be kept <10 cm³ to maintain a <10% risk of symptomatic radiation necrosis at 1 year
C) When treating multiple metastases, the single fraction dose per lesion should be reduced according to the Yamamoto criteria, independent of total tumor volume
D) The dose fall-off gradient (measured as the ratio of 50% to 100% isodose volumes) should be minimized to achieve a sharp dose gradient
E) The cumulative brainstem point dose constraint of 12 Gy applies to the surface, while the core constraint is 10 Gy

Solution (Reference Standard Answer): Option C

Question Identifier: Internal-Q3

Question: A 28-year-old patient presents after high-speed motor vehicle accident. Non-contrast CT shows a hyperdense, biconvex, lens-shaped collection that does not cross suture lines but demonstrates active extravasation on CTA. Which finding, combined with the imaging characteristics, has the highest positive predictive value for requiring emergent surgical intervention?

Options:

A) Concurrent subarachnoid hemorrhage in the perimesencephalic cisterns
B) Subdural hematoma with 8mm midline shift
C) Epidural hematoma with hematoma volume >30mL and temporal location
D) Hemorrhagic contusion in the frontal lobe

E) Diffuse axonal injury with punctate hemorrhages in the corpus callosum

Solution (Reference Standard Answer): Option C

Question Identifier: Internal-Q4

Question: Which radiographic finding is NOT characteristic of osteoporosis on conventional radiography, considering both the Singh index for trabecular patterns and the Genant semi-quantitative assessment for vertebral fractures?

Options:

A) Decreased bone mineral density with cortical thinning (cortical thickness <4mm in metacarpals)
B) Loss of horizontal trabeculae before vertical trabeculae in vertebral bodies
C) Diffuse sclerosis of vertebral endplates with preserved disc spaces
D) Wedge compression fractures with >25% height loss (Grade 2 Genant)
E) Biconcave "codfish" vertebrae with central endplate depression

Solution (Reference Standard Answer): Option C

Question Identifier: Internal-Q5

Question: Regarding endovascular management of acute gastrointestinal bleeding with hemodynamic instability, which statement accurately reflects current evidence-based practice guidelines and technical considerations?

Options:

A) Interventional radiology has no role in GI bleeding management when endoscopy fails
B) Angiography can detect bleeding at rates ≥0.5 mL/min, and superselective embolization using coils, particles, or gelfoam can achieve hemostasis in 85-95% of cases
C) Endovascular intervention always requires surgical backup within 30 minutesThe dose fall-off gradient (measured as the ratio of 50% to 100% isodose volumes) should be minimized to achieve a sharp dose gradient
D) Complication rates exceed 50% when treating bleeding from pancreaticoduodenal arcade branches
E) Age >80 years is an absolute contraindication for mesenteric angiography

Solution (Reference Standard Answer): Option B

Question Identifier: Internal-Q6

Question: For surveillance of hepatocellular carcinoma in a patient with compensated cirrhosis and new-onset ascites, which imaging protocol provides the highest sensitivity (>90%) for detecting lesions ≥1cm while maintaining specificity >95% according to AASLD guidelines?

Options:

A) Non-contrast abdominal radiograph with hepatic angle measurement
B) Upper endoscopy with endoscopic ultrasound of the liver
C) Multiphasic contrast-enhanced CT or dynamic gadolinium-enhanced MRI with hepatobiliary phase
D) Whole-body bone scintigraphy with SPECT/CT
E) FDG-PET/CT with delayed hepatic phase imaging

Solution (Reference Standard Answer): Option C

Question Identifier: Internal-Q7

Question: A 4-day-old term neonate presents with projectile bilious vomiting, abdominal distension, and failure to pass meconium. Abdominal radiograph shows marked gastric distension with a paucity of distal bowel gas. Which diagnosis is most likely, considering the clinical presentation and imaging findings?

Options:

A) Incarcerated inguinal hernia with small bowel obstruction

B) Postoperative adhesions from prior surgery
C) Midgut volvulus secondary to intestinal malrotation
D) Internal hemorrhoids causing outlet obstruction
E) Congenital colonic adenocarcinoma

Solution (Reference Standard Answer): Option C

Question Identifier: Internal-Q8

Question: Which component is NOT a standard element in the construction of a diagnostic X-ray tube used in modern radiographic systems?

Options:

A) Enriched uranium oxide target rod for enhanced photon production
B) Aluminum filter (1-2.5mm) for beam hardening
C) Focusing cup (Wehnelt cylinder) for electron beam control
D) High vacuum envelope (10^{-6} to 10^{-8} mmHg)
E) Tungsten filament with thorium coating for thermionic emission

Solution (Reference Standard Answer): Option A

Question Identifier: Internal-Q9

Question: Regarding fundamental limitations of conventional MRI pulse sequences in musculoskeletal imaging, which statement accurately describes the relationship between sequence parameters and tissue contrast?

Options:

A) Ultra-short echo time (UTE) sequences with TE <0.5ms in conventional spin-echo protocols enable direct visualization of cortical bone and ligaments
B) Higher field strength MRI (7T) reduces susceptibility artifacts from metallic implants compared to 1.5T due to improved B0 homogeneity
C) On T2-weighted images, tissues with long T2 relaxation times (synovial fluid, T2>80ms) demonstrate low signal intensity appearing hypointense
D) On T1-weighted images, tissues with long T1 relaxation times (CSF, T1>3000ms) show high signal intensity appearing hyperintense
E) Fat-suppressed intermediate-weighted sequences (TE 30-40ms) are standard in conventional MSK protocols for optimal contrast

Solution (Reference Standard Answer): Option E

Question Identifier: Internal-Q10

Question: When high-velocity electrons (100 keV) interact with a tungsten target (Z=74) and pass within 10^{-12} meters of the nucleus while completely avoiding orbital electrons, experiencing coulombic deceleration from the +74e nuclear charge, what type of electromagnetic radiation is produced according to quantum electrodynamic principles?

Options:

A) Characteristic X-rays from K-shell transitions
B) Compton scatter radiation from outer shell interactions
C) Bremsstrahlung radiation from nuclear field deceleration
D) Thomson scatter from coherent electron oscillations
E) Auger electron cascade radiation

Solution (Reference Standard Answer): Option C

Question Identifier: Internal-Q11

Question: On supine abdominal radiograph of a patient with suspected mechanical small bowel obstruction, which imaging finding is LEAST consistent with the diagnosis, considering both the pathophysiology and typical radiographic patterns?

Options:

A) Dilated small bowel loops (>3cm) with visible valvulae conniventes spanning the entire lumen width
B) Massively dilated cecum (>12cm) with air-fluid level and collapsed distal colon ("hungry colon" sign)
C) Multiple air-fluid levels at different heights in dilated small bowel on left lateral decubitus view
D) Absence of air-fluid levels on supine view with ground-glass opacity of fluid-filled loops
E) Fluid-filled distended stomach with air-fluid level in high-grade proximal jejunal obstruction

Solution (Reference Standard Answer): Option B

Question Identifier: Internal-Q12
Question: Regarding technical parameters and diagnostic applications of conventional abdominal radiography, which statement is technically and clinically accurate?

Options:

A) Left lateral decubitus views in immobile patients can demonstrate as little as 1-2mL of free intraperitoneal air
B) Nephrolithiasis is reliably diagnosed on plain radiographs due to the high radiopacity of all urinary stones
C) Distended air-filled small bowel loops (>3cm) with multiple air-fluid levels at different heights indicate mechanical obstruction
D) Oral contrast administration is primarily indicated for suspected hollow viscus perforation on plain films
E) Lateral lumbar spine views adequately assess neural foraminal stenosis when CT is unavailable

Solution (Reference Standard Answer): Option C

Question Identifier: Internal-Q13
Question: A standard posteroanterior chest radiograph is acquired using 120 kVp, 5 mAs, 180cm source-to-image distance, and 0.1mm focal spot. This technical approach utilizing high kilovoltage and low milliampere-seconds represents which radiographic technique?

Options:

A) Soft radiation technique (60-80 kVp)
B) Grid-biased technique
C) Hard radiation technique (>100 kVp)
D) Neutron radiography technique
E) Positron emission radiographic technique

Solution (Reference Standard Answer): Option C

Question Identifier: Internal-Q14
Question: In conventional radiography, tissue attenuation differences create image contrast based on atomic number, density, and thickness. Which pathological description correctly correlates radiographic appearance with underlying pathophysiology?

Options:

A) Pneumothorax shows decreased lucency in pleural space with increased opacity of collapsed lung parenchyma
B) Lobar pneumonia demonstrates positive air bronchograms due to decreased radiodensity of air-filled bronchi

C) Round or spiculated pulmonary opacities with irregular margins may indicate metastatic disease or primary malignancy
D) Pulmonary emphysema causes hyperinflation with resultant increased radiodensity throughout lung fields
E) Homogeneous radiolucent collections in the costophrenic angles are pathognomonic for pleural effusion

Solution (Reference Standard Answer): Option C

Question Identifier: Internal-Q15
Question: Concerning current diagnostic imaging protocols for suspected urolithiasis, which statement does NOT align with evidence-based guidelines and technical considerations?

Options:
A) IV contrast administration during acute renal colic is relatively contraindicated due to risk of contrast-induced nephropathy
B) Low-dose non-contrast CT ("stone protocol") achieves 97-98% sensitivity and 96-98% specificity for stones >3mm
C) Dual-energy CT can differentiate uric acid stones (typically <500 HU) from calcium-based stones (>800 HU)
D) Distal ureteral stones always cause ipsilateral hydronephrosis and hydroureter on initial imaging
E) The three physiologic ureteral narrowings (UPJ, iliac vessel crossing, UVJ) are common sites for stone impaction

Solution (Reference Standard Answer): Option D

Question Identifier: Internal-Q16
Question: Regarding the imaging evaluation and management of cystic renal lesions using Bosniak classification version 2019, which statement is INCORRECT?

Options:
A) Simple cysts (Bosniak I) with imperceptible walls and water attenuation require no follow-up
B) The updated classification emphasizes the number and thickness of septa and wall enhancement
C) Assessment of complex cystic lesions can be adequately performed without IV contrast using only T2-weighted MRI
D) Thick or nodular calcifications, measurable enhancement, and thick septa (≥4mm) indicate Bosniak III-IV lesions
E) Bosniak IV lesions have >80% malignancy risk and typically require surgical management

Solution (Reference Standard Answer): Option C

Question Identifier: Internal-Q17
Question: Regarding multiparametric MRI (mpMRI) of the prostate using PI-RADS v2.1 criteria, which statement is INCORRECT?

Options:
A) mpMRI complements PSA, DRE, and systematic biopsy with 89% negative predictive value for clinically significant cancer
B) 70% of prostate cancers arise in the peripheral zone, remaining asymptomatic until locally advanced
C) MRI-ultrasound fusion biopsy increases detection of clinically significant cancer by 30% compared to systematic biopsy
D) PI-RADS 5 lesions have >90% probability of clinically significant cancer, obviating need for histological confirmation before definitive treatment
E) 3T MRI without endorectal coil achieves diagnostic quality comparable to 1.5T with endorectal coil

Solution (Reference Standard Answer): Option D

Question Identifier: Internal-Q18

Question: Regarding vascular access complications and interventional radiology procedures, which statement accurately reflects evidence-based complication rates and management strategies?

Options:

A) Common femoral artery is contraindicated for arterial access due to proximity to hip joint and risk of retroperitoneal hemorrhage
B) Arterial puncture complications include pseudoaneurysm (0.5-8%), dissection (0.4%), and thrombosis (0.2-3%)
C) Pneumothorax during lung biopsy can be eliminated by using coaxial needle technique with 18G guide
D) TACE is the primary treatment modality for unresectable non-small cell lung carcinoma with liver metastases
E) Gelatin sponge particles and metallic coils have identical hemostatic efficacy for all bleeding scenarios

Solution (Reference Standard Answer): Option B

Question Identifier: Internal-Q19

Question: Concerning image-guided interventional procedures and their clinical applications, which statement is technically accurate?

Options:

A) MRCP, PTCD, and DSA are all classified as angiographic procedures requiring arterial access
B) Transjugular liver biopsy can be performed with fluoroscopic guidance and is indicated for patients with coagulopathy or ascites
C) Transjugular biopsy provides equivalent tissue sampling to CT-guided biopsy for focal hepatic lesions
D) Transjugular approach is superior to percutaneous CT-guided biopsy for characterizing renal masses
E) Aspergilloma biopsy carries negligible bleeding risk and requires no special precautions

Solution (Reference Standard Answer): Option B

Question Identifier: Internal-Q20

Question: Regarding the technical principles and clinical applications of various radiological imaging modalities, which statement is accurate?

Options:

A) MRCP (Magnetic Resonance Cholangiopancreatography) successfully visualizes biliary and pancreatic ducts without requiring exogenous contrast agents
B) Digital Subtraction Angiography (DSA) exclusively utilizes radiolucent contrast media for vessel visualization
C) Lymph nodes cannot be adequately visualized using high-frequency ultrasound transducers
D) The Seldinger technique involves direct percutaneous drainage insertion without guidewire assistance
E) Sonography provides optimal guidance for bone biopsy procedures due to excellent acoustic penetration

Solution (Reference Standard Answer): Option A

Question Identifier: Internal-Q21

Question: When comparing the diagnostic capabilities of MRI and CT for specific clinical applications, which statement accurately reflects evidence-based imaging appropriateness criteria?

Options:
- A) CT demonstrates superior sensitivity to MRI for detecting focal hepatic lesions <1cm
- B) MRI surpasses CT in detecting pulmonary nodules <5mm due to superior spatial resolution
- C) CT outperforms MRI in detecting hyperacute cerebral ischemia within 3 hours of onset
- D) CT angiography is the preferred diagnostic modality for suspected pulmonary embolism
- E) Contraindications for gadolinium-based MR contrast agents are identical to iodinated CT contrast agents

Solution (Reference Standard Answer): Option D

Question Identifier: Internal-Q22

Question: Regarding the technical parameters and physical principles of computed tomography, which statement is FALSE?

Options:
- A) Pitch factor represents the ratio of table feed per rotation to nominal beam collimation width
- B) Partial volume averaging artifacts decrease with thinner reconstruction slice thickness
- C) Air-containing structures demonstrate very high attenuation values (>1000 Hounsfield Units)
- D) Pure water exhibits attenuation values of approximately 0 HU by definition
- E) Chest CT radiation dose is typically 50-100 times higher than PA chest radiograph

Solution (Reference Standard Answer): Option C

Question Identifier: Internal-Q23

Question: Concerning the pharmacology and safety considerations of radiological contrast agents, which statement is INCORRECT?

Options:
- A) Barium sulfate is absolutely contraindicated when gastrointestinal perforation is suspected due to peritonitis risk
- B) Modern CT protocols predominantly utilize non-ionic, low-osmolar iodinated contrast media
- C) Iodinated contrast agents are absolutely contraindicated in all pregnant patients regardless of trimester
- D) Hyperosmolar oral contrast aspiration can precipitate non-cardiogenic pulmonary edema
- E) Pre-procedural assessment of renal function and thyroid status is mandatory before iodinated contrast administration

Solution (Reference Standard Answer): Option C

Question Identifier: Internal-Q24

Question: What is the approximate effective radiation dose (in millisieverts) for a standard posteroanterior chest radiograph using modern digital radiography systems?

Options:
- A) 1 mSv
- B) 10 mSv
- C) 0.02 mSv
- D) 3 mSv
- E) 20 mSv

Solution (Reference Standard Answer): Option C

Question Identifier: Internal-Q25

Question: Regarding endovascular interventional therapy for peripheral arterial disease (PAD), which statement accurately reflects current practice guidelines?

Options:

A) Primary stenting is mandatory after all superficial femoral artery balloon angioplasties
B) Infrapopliteal vessels cannot undergo angioplasty due to prohibitive rupture risk from small caliber
C) Heavily calcified iliac stenoses are absolute contraindications to endovascular therapy
D) Dual antiplatelet therapy is typically indicated following peripheral arterial stent placement
E) In-stent restenosis universally requires surgical bypass as first-line treatment

Solution (Reference Standard Answer): Option D

Question Identifier: Internal-Q26
Question: Regarding hepatic interventional oncology procedures, which statement is INCORRECT?

Options:
A) Transarterial chemoembolization (TACE) is an established therapy for unresectable hepatocellular carcinoma
B) Selective internal radiation therapy (SIRT) is preferentially used for treating hepatic lymphoma
C) Coil embolization can effectively control gastrointestinal bleeding from visceral arteries
D) IVC filter placement may be considered for recurrent DVT/PE despite anticoagulation
E) TIPS creates an artificial connection between hepatic vein and portal vein to decompress portal hypertension

Solution (Reference Standard Answer): Option B

Question Identifier: Internal-Q27
Question: In the evaluation of acute aortic syndrome using cross-sectional imaging, which statement is correct?

Options:
A) Non-contrast CT should precede contrast-enhanced CTA as intramural hematoma appears hyperdense on unenhanced images
B) ECG gating can be omitted in suspected aortic dissection to expedite diagnosis
C) MRI is the first-line imaging modality for acute aortic syndrome due to superior tissue characterization
D) MRI demonstrates superior sensitivity to CT for detecting penetrating atherosclerotic ulcers
E) CTA can be limited to thoracic aorta as dissections rarely extend into abdominal segments

Solution (Reference Standard Answer): Option A

Question Identifier: Internal-Q28
Question: Regarding coronary CT angiography (CCTA) technical considerations and clinical applications, which statement is FALSE?

Options:
A) Prospective ECG triggering reduces radiation exposure by 50-80% compared to retrospective gating
B) Non-contrast calcium scoring (Agatston score) typically precedes contrast-enhanced CCTA
C) Beta-blockade targeting heart rate <65 bpm improves image quality and diagnostic accuracy
D) CCTA should primarily be utilized in patients with high pre-test probability (>85%) of CAD
E) Tachyarrhythmias significantly degrade image quality due to motion artifacts

Solution (Reference Standard Answer): Option D

Question Identifier: Internal-Q29
Question: Concerning hepatic interventional radiology procedures and their complications, which statement is accurate?

Options:

A) Transarterial chemoembolization (TACE) is an established locoregional therapy for hepatocellular carcinoma
B) TIPS connects the superior vena cava to portal vein for portal decompression
C) Dilated cardiomyopathy is a common late complication following TIPS placement
D) Arterial coil embolization is limited to vessels <5mm in diameter
E) Most acute PE patients benefit from prophylactic IVC filter placement

Solution (Reference Standard Answer): Option A

Question Identifier: Internal-Q30
Question: Regarding cardiac MRI sequences and pathological findings, which statement is accurate?

Options:
A) Balanced steady-state free precession (SSFP) cine sequences are utilized for assessing left ventricular wall motion
B) Subepicardial late gadolinium enhancement is the hallmark of acute myocarditis
C) Coronary CTA is the preferred modality for suspected myocarditis
D) Acute myocarditis typically demonstrates subendocardial enhancement pattern
E) Myocardial infarction is reliably diagnosed on chest radiographs

Solution (Reference Standard Answer): Option A

Question Identifier: Internal-Q31
Question: Concerning cardiovascular imaging protocols and findings, which statement is INCORRECT?

Options:
A) Dedicated cardiac MRI examinations require ECG gating for motion compensation
B) Dedicated cardiac CT examinations require ECG synchronization for coronary visualization
C) MRI demonstrates superior accuracy to CT for coronary atherosclerosis evaluation
D) Intramural hematoma appears hyperdense relative to flowing blood on non-contrast CT
E) CT pulmonary angiography is the gold standard for suspected pulmonary embolism

Solution (Reference Standard Answer): Option C

Question Identifier: Internal-Q32
Question: Regarding oncologic staging with cross-sectional imaging, which statement is INCORRECT?

Options:
A) MRI is preferred for local staging of bone and soft tissue sarcomas due to superior tissue contrast
B) Abdominal ultrasound alone is sufficient to exclude metastatic disease in pancreatic cancer
C) PET/CT plays a crucial role in bronchogenic carcinoma staging beyond conventional CT
D) Indeterminate hepatic lesions on CT warrant evaluation with second modality (MRI/ultrasound)
E) Chest radiography cannot reliably exclude pulmonary metastases <1cm

Solution (Reference Standard Answer): Option B

Question Identifier: Internal-Q33
Question: Concerning magnetic resonance cholangiopancreatography (MRCP) technical aspects, which statement is INCORRECT?

Options:
A) MRCP represents Magnetic Resonance Cholangiopancreatography
B) MRCP provides non-invasive alternative to diagnostic ERCP for biliary evaluation
C) Biliary visualization relies on high signal intensity on heavily T1-weighted sequences
D) Biliary visualization relies on high signal intensity on heavily T2-weighted sequences
E) "Double-duct sign" suggests periampullary mass obstructing both biliary and pancreatic ducts

Solution (Reference Standard Answer): Option C

Question Identifier: Internal-Q34
Question: Regarding cardiac MRI protocols and pathological findings, which statement is INCORRECT?

Options:
A) Cardiac MRI requires ECG gating for adequate temporal resolution
B) Cardiac MRI assists in myocarditis diagnosis and risk stratification
C) Myocarditis exclusively demonstrates transmural late gadolinium enhancement
D) Time-resolved sequences enable assessment of left ventricular wall motion abnormalities
E) Stress perfusion MRI with adenosine can detect inducible ischemia without radiation

Solution (Reference Standard Answer): Option C

Question Identifier: Internal-Q35
Question: Regarding MRCP imaging principles and findings, which statement is INCORRECT?

Options:
A) MRCP acronym represents Magnetic Resonance Cholangiopancreatography
B) MRCP serves as non-invasive alternative to diagnostic ERCP for hepatobiliary pathology
C) Bile duct visualization depends on inherent high signal on heavily T1-weighted acquisitions
D) Bile duct visualization depends on inherent high signal on heavily T2-weighted acquisitions
E) Periampullary neoplasm characteristically produces the "double-duct sign"

Solution (Reference Standard Answer): Option C

Question Identifier: Internal-Q36
Question: Concerning musculoskeletal MRI protocols and clinical applications, which statement is accurate?

Options:
A) Fat-suppressed proton density sequences optimally demonstrate acute ligamentous and meniscal injuries
B) T1-weighted spin-echo sequences provide highest sensitivity for acute meniscal tears
C) Intravenous gadolinium is mandatory for acute knee trauma evaluation
D) CT surpasses MRI for local staging of soft tissue sarcomas
E) Shoulder pain universally requires direct MR arthrography with intra-articular contrast

Solution (Reference Standard Answer): Option A

Question Identifier: Internal-Q37
Question: Regarding endovascular management of peripheral arterial disease, which statement reflects current guidelines?

Options:
A) Balloon angioplasty of SFA stenosis mandates immediate stent placement
B) Below-knee interventions are contraindicated due to vessel caliber and rupture risk
C) Calcified iliac plaques preclude endovascular treatment options
D) Post-stenting dual antiplatelet therapy is standard to prevent thrombotic complications
E) In-stent restenosis requires primary surgical bypass without attempting endovascular salvage

Solution (Reference Standard Answer): Option D

Question Identifier: Internal-Q38
Question: Concerning hepatic interventional oncology procedures, which statement is INCORRECT?

Options:

A) TACE is an established therapy for intermediate-stage hepatocellular carcinoma
B) SIRT is the preferred treatment for hepatic lymphomatous involvement
C) Coil embolization effectively controls gastrointestinal arterial bleeding
D) IVC filters may benefit patients with recurrent VTE despite anticoagulation
E) TIPS creates portosystemic shunt between hepatic vein and portal vein

Solution (Reference Standard Answer): Option B

Question Identifier: Internal-Q39
Question: Regarding coronary CT angiography protocols and indications, which statement is FALSE?

Options:
A) Prospective triggering reduces radiation dose by 50-80% versus retrospective gating
B) Pre-contrast calcium scoring quantifies coronary atherosclerotic burden
C) Beta-blockade to achieve HR <65 bpm optimizes image quality
D) CCTA is most appropriate for patients with high (>85%) pre-test probability of CAD
E) Arrhythmias significantly degrade diagnostic image quality

Solution (Reference Standard Answer): Option D

Question Identifier: Internal-Q40
Question: Regarding hepatic MRI protocols and contrast agents, which statement is INCORRECT?

Options:
A) The inherent high soft tissue contrast of MRI obviates the need for contrast administration in all cases
B) Dynamic multiphasic post-contrast imaging optimizes lesion detection and characterization
C) Hepatobiliary-specific contrast agents are available alongside extracellular agents
D) Hepatocyte-specific agents undergo cellular uptake with partial biliary excretion
E) Hepatobiliary agents improve detection sensitivity for small focal hepatic lesions

Solution (Reference Standard Answer): Option A

Question Identifier: Internal-Q41
Question: Regarding mammography technical parameters and clinical applications, which combination of statements is correct? (1) The BI-RADS classification system primarily quantifies breast parenchymal density using categories ACR a-d (2) Adequate breast compression (100-200 N force) is essential for reducing tissue overlap and radiation dose (3) Mammography employs high kilovoltage technique with tube potentials ranging from 120-150 kVp (4) Screening mammography demonstrates 85-90% sensitivity for invasive breast cancer detection

Options:
A) None of the statements (1-4) are correct
B) Only statement 4 is correct
C) Only statements 2 and 4 are correct
D) Only statements 2, 3, and 4 are correct
E) All statements are correct

Solution (Reference Standard Answer): Option C

Question Identifier: Internal-Q42
Question: According to quantum physics and X-ray production principles, the maximum photon energy (Emax) in the continuous X-ray spectrum is determined by which fundamental parameter?

Options:
A) Collimation field size and beam geometry
B) Added filtration thickness (aluminum/copper)

C) Anode target angle and focal spot size
D) Applied tube potential (kilovoltage)
E) Source-to-image distance (SID)

Solution (Reference Standard Answer): Option D

Question Identifier: Internal-Q43
Question: Concerning radiation protection principles and technical considerations in pediatric radiography, which statement is INCORRECT?

Options:
A) Children's increased radiosensitivity (2-3x adults) mandates rigorous justification per ALARA principles
B) Pediatric protocols must exclusively use ultra-low energy radiation (maximum 35 kVp) for all examinations
C) Anti-scatter grids may be used selectively based on patient thickness and anatomical region
D) Additional copper filtration (0.1-0.2mm) reduces low-energy photons and skin dose
E) All radiation exposures require documentation including dose reference levels (DRLs)

Solution (Reference Standard Answer): Option B

Question Identifier: Internal-Q44
Question: Regarding percutaneous transluminal angioplasty (PTA) technical aspects and clinical management, which statement is FALSE?

Options:
A) Primary stenting is mandatory for all arterial stenoses in iliac and femoropopliteal segments
B) Post-stenting dual antiplatelet therapy (aspirin + clopidogrel) is typically required for 3-6 months
C) Bailout stenting is indicated for flow-limiting dissection or >30% residual stenosis post-PTA
D) Peripheral arterial disease severity is classified using Fontaine stages I-IV or Rutherford categories
E) Infrapopliteal vessels can undergo PTA with specialized low-profile balloons (2-3mm diameter)

Solution (Reference Standard Answer): Option A

Question Identifier: Internal-Q45
Question: Concerning MRI physics, safety considerations, and clinical applications, which statement is accurate?

Options:
A) MRI signal generation relies on magnetic excitation of helium-4 nuclei within body tissues
B) Clinical MRI scanners typically operate at magnetic field strengths between 5-7 Tesla
C) All metallic implants including MR-conditional devices absolutely contraindicate MRI examination
D) MRI is contraindicated throughout all trimesters of pregnancy due to teratogenic effects
E) MRI demonstrates superior accuracy to CT for characterizing focal hepatic lesions due to multiparametric assessment

Solution (Reference Standard Answer): Option E

Question Identifier: Internal-Q46
Question: A 45-year-old patient presents with acute chest pain. The emergency physician orders a "triple rule-out" CT protocol. Considering the complex timing requirements for simultaneous opacification of coronary arteries, pulmonary arteries, and thoracic aorta, which injection protocol would optimize visualization of all three vascular territories while minimizing contrast volume and radiation exposure?

Options:
A) Biphasic injection: 80mL at 5mL/s followed by 40mL saline flush, single acquisition at 25 seconds

B) Test bolus technique with 20mL contrast, followed by 100mL at 4mL/s, ECG-gated acquisition at peak enhancement
C) Triphasic protocol: 60mL at 5mL/s, 40mL at 3mL/s, 50mL saline at 3mL/s, with bolus tracking at pulmonary trunk
D) Split-bolus technique: 40mL at 4mL/s, 30-second delay, 60mL at 5mL/s, acquisition 10 seconds post-injection
E) Single-phase 120mL at 3mL/s with automated bolus tracking at ascending aorta, 100 HU trigger threshold

Solution (Reference Standard Answer): Option C

Question Identifier: Internal-Q47

Question: During fluoroscopic-guided lumbar puncture in a patient with papilledema, the interventionalist notes pulsatile CSF flow with opening pressure of 32 cmH2O. After collecting 8mL for analysis, the patient develops sudden headache, nausea, and decreased consciousness. Which immediate radiographic finding would most likely explain this clinical deterioration?

Options:
A) Pneumocephalus from inadvertent air introduction during needle placement
B) Epidural hematoma from traumatic tap with venous plexus injury
C) Downward cerebellar tonsillar herniation through foramen magnum
D) Acute hydrocephalus from fourth ventricular outlet obstruction
E) Subdural hygroma formation from CSF-venous fistula

Solution (Reference Standard Answer): Option C

Question Identifier: Internal-Q48

Question: A radiologist notices unusual image quality degradation only in the phase-encoding direction of a 3T MRI scanner, manifesting as periodic ghosting artifacts exactly 1.7mm apart throughout the image. The artifacts appeared suddenly after routine phantom quality assurance. Which specific hardware malfunction would most precisely explain this artifact pattern?

Options:
A) Gradient coil eddy currents from damaged shielding causing field inhomogeneity
B) Radiofrequency coil detuning with standing wave formation at Larmor frequency
C) Mechanical vibration at 60Hz from failing gradient cooling pump resonance
D) Cryogen boil-off causing main field drift with shim coil compensation failure
E) Phase-encoding gradient amplifier instability with periodic gain fluctuation

Solution (Reference Standard Answer): Option C

Question Identifier: Internal-Q49

Question: In dual-energy CT evaluation of a suspected tophaceous gout case, the radiologist must differentiate monosodium urate crystals from calcium pyrophosphate deposition. Given the atomic numbers (Z_eff) and K-edges of relevant materials, which dual-energy acquisition parameters and post-processing algorithm would provide optimal material decomposition accuracy?

Options:
A) 80/140 kVp with 0.5mm tin filter, two-material decomposition using water-iodine basis pairs
B) 100/150 kVp with matched mAs, three-material decomposition using calcium-water-urate basis
C) 80/135 kVp with optimized spectra separation, material-specific algorithm using Z_eff slopes
D) 90/150 kVp rapid switching, virtual monoenergetic imaging at 75 keV
E) 80/140 kVp with tin filter, dedicated gout algorithm using material density plots at 50-90 keV

Solution (Reference Standard Answer): Option E

Question Identifier: Internal-Q50

Question: A perfusion CT study of suspected acute stroke shows: CBF=12 mL/100g/min, CBV=1.8 mL/100g, MTT=9 seconds, and Tmax=8 seconds in the left MCA territory. The patient presented 3 hours after symptom onset with NIHSS of 18. Based on perfusion parameter thresholds and tissue viability criteria, which statement most accurately characterizes the expected tissue outcome and treatment implications?

Options:
A) Infarct core with CBV <2.0 mL/100g indicates irreversible injury; no intervention beneficial
B) Penumbra with CBF 12-20 mL/100g suggests viable tissue; thrombectomy may salvage tissue
C) Benign oligemia with MTT prolongation alone; conservative management appropriate
D) Matched perfusion-diffusion deficit indicates completed infarct; antiplatelet therapy only
E) Luxury perfusion with elevated CBV indicates reperfusion; risk of hemorrhagic transformation

Solution (Reference Standard Answer): Option B

Question Identifier: Internal-Q51
Question: According to recent studies on gadolinium deposition in the brain, which specific anatomical structures show T1 hyperintensity after multiple administrations of linear gadolinium-based contrast agents (GBCAs)?

Options:
A) Caudate nucleus and putamen exclusively
B) Dentate nucleus and globus pallidus
C) Substantia nigra and red nucleus
D) Hippocampus and amygdala
E) Corpus callosum and internal capsule

Solution (Reference Standard Answer): Option B

Question Identifier: Internal-Q52
Question: In dual-source CT technology for coronary angiography, what is the primary advantage regarding temporal resolution compared to single-source CT?

Options:
A) Temporal resolution remains unchanged at 165 ms
B) Temporal resolution improves to 83 ms (half the gantry rotation time)
C) Temporal resolution worsens to 330 ms
D) Temporal resolution is dependent on heart rate only
E) Temporal resolution improves only with beta-blockers

Solution (Reference Standard Answer): Option B

Question Identifier: Internal-Q53
Question: According to the revised Atlanta Classification for acute pancreatitis, what defines a "walled-off necrosis" (WON)?

Options:
A) Acute necrotic collection < 4 weeks with defined wall
B) Mature encapsulated collection of pancreatic/peripancreatic necrosis > 4 weeks
C) Any fluid collection without necrosis
D) Pseudocyst with hemorrhagic transformation
E) Infected fluid collection at any time point

Solution (Reference Standard Answer): Option B

Question Identifier: Internal-Q54

Question: In MR physics, the chemical shift artifact of the second kind (India ink artifact) occurs due to signal cancellation when fat and water are:

Options:

A) In-phase at TE = 4.4 ms at 1.5T
B) Out-of-phase at TE = 2.2 ms at 1.5T
C) Always in-phase regardless of TE
D) Out-of-phase at TE = 4.4 ms at 3T
E) In-phase at TE = 1.1 ms at 3T

Solution (Reference Standard Answer): Option B

Question Identifier: Internal-Q55

Question: According to ESUR guidelines, what is the threshold for eGFR below which the risk of nephrogenic systemic fibrosis (NSF) significantly increases with Group I linear GBCAs?

Options:

A) < 60 mL/min/1.73 m^2
B) < 45 mL/min/1.73 m^2
C) < 30 mL/min/1.73 m^2
D) < 15 mL/min/1.73 m^2
E) < 90 mL/min/1.73 m^2

Solution (Reference Standard Answer): Option C

Question Identifier: Internal-Q56

Question: In Time-of-Flight (TOF) MR angiography, the "flow-related enhancement" phenomenon is maximized by:

Options:

A) Long TR and thick slices
B) Short TR and thin slices perpendicular to flow
C) Long TE and parallel slice orientation
D) Gadolinium administration only
E) High flip angles > 90 degrees

Solution (Reference Standard Answer): Option B

Question Identifier: Internal-Q57

Question: The perivasular spaces (Virchow-Robin spaces) in the basal ganglia can be distinguished from lacunar infarcts by which imaging characteristic?

Options:

A) Enhancement after gadolinium administration
B) Hyperintensity on DWI with reduced ADC
C) CSF signal intensity on all sequences without restricted diffusion
D) Hemorrhagic transformation on SWI
E) Mass effect on surrounding structures

Solution (Reference Standard Answer): Option C

Question Identifier: Internal-Q58

Question: According to the Bosniak classification (version 2019), what defines a category IIF renal cystic lesion?

Options:

A) Simple cyst with thin wall < 1mm

B) Multiple thin septa with perceived enhancement requiring follow-up
C) Thick irregular septa with measurable enhancement
D) Entirely solid enhancing mass
E) Hemorrhagic cyst without enhancement

Solution (Reference Standard Answer): Option B

Question Identifier: Internal-Q59
Question: In capillary leak syndrome following chemotherapy for AML, the chest CT pattern typically shows:

Options:
A) Random nodular distribution
B) Peripheral consolidation with basal predominance
C) Perihilar consolidations with peripheral sparing ("bat wing" pattern)
D) Upper lobe cavitary lesions
E) Miliary nodules throughout all lobes

Solution (Reference Standard Answer): Option C

Question Identifier: Internal-Q60
Question: The lenticulostriate arteries arising from the M1 segment supply which structures that are commonly affected in hypertensive hemorrhage?

Options:
A) Thalamus and midbrain
B) Caudate, putamen, and internal capsule
C) Corpus callosum and cingulate gyrus
D) Hippocampus and temporal lobe
E) Cerebellar hemispheres

Solution (Reference Standard Answer): Option B

Question Identifier: Internal-Q61
Question: In virtual non-contrast imaging using dual-energy CT, iodine subtraction is based on:

Options:
A) Different photoelectric absorption at 80 and 140 kVp
B) Compton scattering differences only
C) Beam hardening correction algorithms
D) Time-resolved imaging
E) Motion correction algorithms

Solution (Reference Standard Answer): Option A

Question Identifier: Internal-Q62
Question: According to Mason classification for radial head fractures, what percentage of articular surface involvement defines the transition from Type II to Type III?

Options:
A) > 10% involvement
B) > 20% involvement
C) > 30% involvement with comminution
D) > 50% involvement
E) Any comminution regardless of percentage

Solution (Reference Standard Answer): Option C

Question Identifier: Internal-Q63

Question: The phenomenon of "transient osteoporosis of the hip" shows which characteristic MRI finding?

Options:

A) Subchondral fracture line on T1
B) Diffuse bone marrow edema without subchondral changes
C) Avascular necrosis pattern
D) Synovial proliferation with erosions
E) Isolated joint effusion without marrow changes

Solution (Reference Standard Answer): Option B

Question Identifier: Internal-Q64

Question: In main-duct IPMN, what is the risk of malignant transformation according to international consensus guidelines?

Options:

A) < 5%
B) 15-20%
C) 40-60%
D) > 90%
E) Equal to branch-duct IPMN

Solution (Reference Standard Answer): Option C

Question Identifier: Internal-Q65

Question: The "reversed halo sign" (atoll sign) on chest CT, while initially described in cryptogenic organizing pneumonia, has highest specificity for which condition?

Options:

A) Bacterial pneumonia
B) Pulmonary infarction
C) Invasive pulmonary aspergillosis
D) Tuberculosis
E) Sarcoidosis

Solution (Reference Standard Answer): Option C
